# The influencing factors and spillover effects of interprovincial agricultural carbon emissions in China

**DOI:** 10.1371/journal.pone.0240800

**Published:** 2020-11-04

**Authors:** Weidong Chen, Yufang Peng, Guanyi Yu

**Affiliations:** 1 College of Management and Economics, Tianjin University, Tianjin, China; 2 School of Architecture, Tianjin University, Tianjin, China; Institute for Advanced Sustainability Studies, GERMANY

## Abstract

Agricultural carbon emissions have become the constraints of agricultural low-carbon and circular economy development in China. China’s agricultural production faces severe pressures and challenges in agricultural carbon reduction. In this paper, we take observation for the 31 provinces in china from 1997 to 2017, to explore the influencing factors and spatial spillover effects of agricultural by estimating spatial panel data models. The results show that China’s agricultural carbon emissions will continue to increase in the future, because the growth of per capita gross domestic product (GDP) is the main driving force to accelerate the growth of agricultural carbon emissions, but the agricultural input factors will help to reduce the growth of carbon emissions. Moreover, it is proved that economic factors and agricultural input factors have direct effects and spatial spillover effects on agricultural carbon emissions except for agricultural environmental factors. In the short term, strengthening environmental protection may bring some pressure to the economic development of some places, but to achieve high-quality development, we must fundamentally solve the problem of environmental pollution. The conclusion provides important enlightenment and scientific basis for formulating effective policies to curb the growth of CO_2_ emissions in China.

## Introduction

The global warming caused by greenhouse gases has become the biggest threat to human beings in the future. In 2018, global carbon dioxide emissions increased by 1.7%, the total emissions reached the highest level in history (33.1 billion tons). Since 2017, China has become the second largest source of greenhouse gases. Agricultural carbon emission is accounts for 17% of the national total [1]. If we do not take effective measures to reduce emissions, it is predicted that agricultural greenhouse gases will increase by 30% in 2050 [2]. Agriculture sector is not only a major source of greenhouse gas emissions, but also, as one of the most vulnerable sectors to climate change. Agricultural emission reduction is a binding goal incorporated into the national economy, social development and long-term planning [3]. Agricultural carbon emission reduction is an important way to improve the ability of agriculture to cope with climate change, and an important link to realize the sustainable development of agriculture [4]. How to control the carbon emission caused by agriculture sector is a complex and important issue.

China’s agriculture sector faces the dual objectives of ensuring food security and reducing carbon emissions. In agricultural production, it is necessary to improve the output efficiency of unit agricultural input, on the other hand, reducing the carbon consumption of unit output is also essential [5]. The agricultural sector is considered to be the most difficult sector to achieve carbon emission reduction goal. As the largest agricultural country, China gains the largest agricultural production while also generating the largest carbon emissions. In recent years, in order to ensure food security and increase agricultural production, the use of chemical fertilizer, pesticide and agricultural machinery has increased year by year. Moreover, due to the increase of population and economic growth, the growth of agricultural carbon dioxide emissions in China has been accelerated.

At present, the research on agricultural carbon emission mainly focuses on the calculation of agricultural carbon emission and its driving factors [6–9]. Agricultural carbon emission mainly comes from farmland utilization, paddy field, livestock intestinal fermentation and manure management. Among them, agricultural land use carbon emissions (farmland ecosystem carbon emissions), accounted for 34.29% of the total agricultural carbon emissions [10]. In order to explore the path of agricultural sustainable development, scholars have done a lot of research on the influencing factors of agricultural carbon emissions, it involves different countries and regions. However, the study mainly focused on the cross section data, focusing on IPAT model of STIRPAT model environmental Kuznets curve, LMDI decomposition model to analysis of influence factors in the whole, ignoring the due to the regional agricultural production and operation of the geographical condition convergence, population flows, mode of production and technology promotion and diffusion, regional agricultural production and operation activities affect each other and function [1,5,9,11]. At present, there are also a few literatures using short-term panel data to quantitatively analyze the spatial characteristics and driving factors of agricultural carbon emissions in China. However, it mainly focuses on the analysis of the influencing factors in the provincial level, or studies on a specific industry, such as vegetable planting, fruit tree planting, etc. [4,7,12,13].

The commonly decomposition model of carbon emission factors includes LMDI decomposition method, Kaya identities and STRIPAT model [1,9,10]. Most of study found that, GDP of planting industry, GDP of agriculture, regional GDP, total regional population and total rural population, agricultural production efficiency, agricultural industrial structure, industrial structure, regional economic development level, urbanization all of the factors have reflected the influencing factors of agricultural carbon emissions [4,11,13]. Based on the above literature analysis, some appropriate variables were selected in this paper, and the appropriate deformation of variables was carried out through statistical analysis.

Research on spatial characteristics shows that China’s carbon dioxide emissions are increasing, with a gradual spatial agglomeration effect. In addition, the study also found that carbon dioxide emissions varied widely between cities, and show a trend of increasing year by year. The conclusion emphasizes how the importance of spatial characteristics of CO_2_ emission between cities for emission reduction [14–17]. But the analysis of the agricultural carbon emissions’ influencing factors mainly focuses on economy and population [6,14]. Few scholars have explored the implicit constraints of agricultural economic and social development from the perspective of input, neglecting technological progress, climate change, and the phenomenon of increasing agricultural carbon emissions due to excessive pursuit of agricultural output.

This study attempts to build a spatial panel model, through panel data of 31 provinces from 1999 to 2017, to explore the influencing factors of agriculture carbon emissions in China and its spatial spillover effect. The study focuses on the following problems. Firstly, it proposed a method to obtain agricultural carbon emission data at the micro level. Then, from the perspective of space, we analyze the distribution of China’s agricultural structure, economic growth, technological level, agricultural investment, agricultural carbon emissions, and the spatial auto-correlation of variables; Finally, this paper further studies the spatial spillover effect of agricultural carbon emission factors on agricultural carbon emission. There are important theoretical and practical significance for reducing cross regional carbon emissions and promoting economic cooperation addressing the above mentioned issues.

The framework of this paper is as follows. The next section describes the methods and data used in model estimation. In Section 3, the research results are given, and the influencing factors and spatial spillover effects are explained. Finally, the paper gives the conclusion and policy enlightenment.

## Methodology and data

### Calculation of agricultural carbon emissions

**Fig 1:**
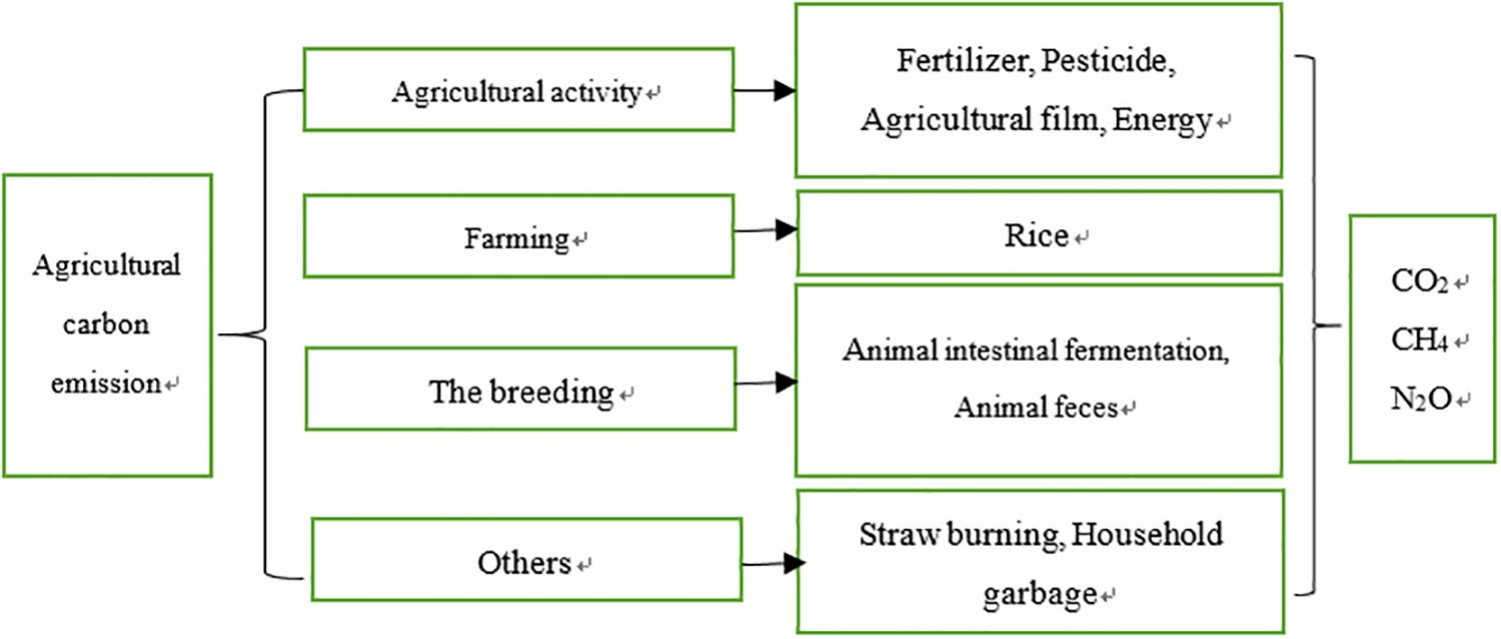


When studying the influencing factors of agricultural carbon emissions, it is the first issue for researchers to identify the sources of agricultural carbon emissions. According to the previous studies (as shown in Fig 1). China’s agricultural carbon emissions involve agricultural activities, planting and aquaculture. Carbon dioxide is mainly emitted from agricultural activities, especially factory agriculture and production mode [3,5,18]. In the process of agricultural production, such as irrigation, the use of chemical fertilizer and energy, most aspects of agricultural production will generate a lot of carbon emissions. In the paper, we focus on four key points to explore the impacts of agricultural input on carbon emissions in agricultural.

According to IPCC (2007), agricultural carbon emissions can be calculated by formula (1):

$$C=\sum_{i=1}^{n}C_{i}=\sum_{i=1}^{n}T_{i}\delta_{i}$$ (1)

Where C stands for agricultural carbon emission, *C*_*i*_ is the different provinces of carbon emission, *T*_*i*_ is the different sources of carbon emission, and *δ*_*i*_ is the coefficient of different carbon emission sources.

In this paper, agricultural carbon emission sources are divided into three parts: carbon emission from agricultural production activities, CO_2_ emission from rice crop production, and CO_2_ emission from livestock and poultry.

Through Eq (2), the carbon emission of agricultural production activities is estimated:

$$C_{a}=\sum_{i=1}^{n}W_{i}\varepsilon_{i}$$ (2)

Where, *C*_*a*_ is the carbon emission from agricultural production activities, and *W*_*i*_ is the consumption of fertilizer, pesticide, agricultural plastic film and agricultural diesel oil, and the coefficient of *ε*_*i*_ is shown in Table 1.

**Table 1:** Carbon emission coefficient of major agricultural production activities.

Carbon Emission Source	Carbon Emission Factor	Literature sources
**Fertilizer**	0.8956 kg c/kg	[19]
**Pesticide**	4.9341 kg c/kg	[20]
**Agricultural Irrigation**	266.48 kg c/hm^2^	[19]
**Agricultural Diesel**	0.5927 kg c/kg	IPCC (2007)
**Agricultural Plastic Film**	0.8956 kg c/kg	[21]

In the calculation of rice planting and animal husbandry. First we calculate methane emissions, and then we convert it to CO_2_ equivalent.

Methane produced by rice cultivation is the main source of carbon emission. Rice mainly involves early season rice, semilate rice and late season rice. In this paper, rice is selected as the research object, and the emission coefficient of rice is referred to Wen et al. [14], the carbon emission generated in difference of rice growth is calculated by formula (3).

$$C_{r}=\sum_{i=1}^{n}N_{i}\mu_{i}$$ (3)

CH_4_ emission from animal husbandry mainly comes from the formation of animal feces and fermentation of stomach. The main animal husbandry in China includes cattle, mules, horses, camels, donkeys, pigs, sheep and poultry. According to IPCC (2007) and [22] different animal husbandry emission coefficients, CH_4_ emissions of 8 animal husbandry are estimated by formula (4) (see Table 2).

$$C_{r}=\sum_{i=1}^{n}S_{i}\gamma_{i}$$ (4)

Where, *S*_*i*_ is the quantity of different livestock and *γ*_*i*_ is the emission coefficient of corresponding livestock.

**Table 2:** CH_4_ emission coefficient of various livestock (kg/head).

Sources	Poultry	Sheep	Pig	Donkey	Camel	Horse	Mule	Cow
**Intestinal fermentation**	0	5	1	10	46	18	10	59.7
**Manure**	0.02	0.16	3.5	0.9	1.92	1.64	0.9	8.75

Sources: IPCC (2007) and Chen [22].

### Descriptive analysis of data

**Fig 2:**
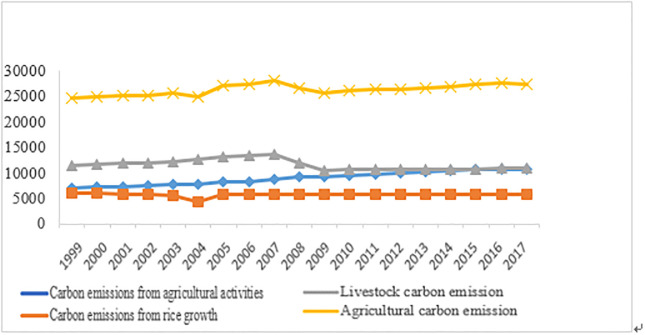


**Fig 3:**
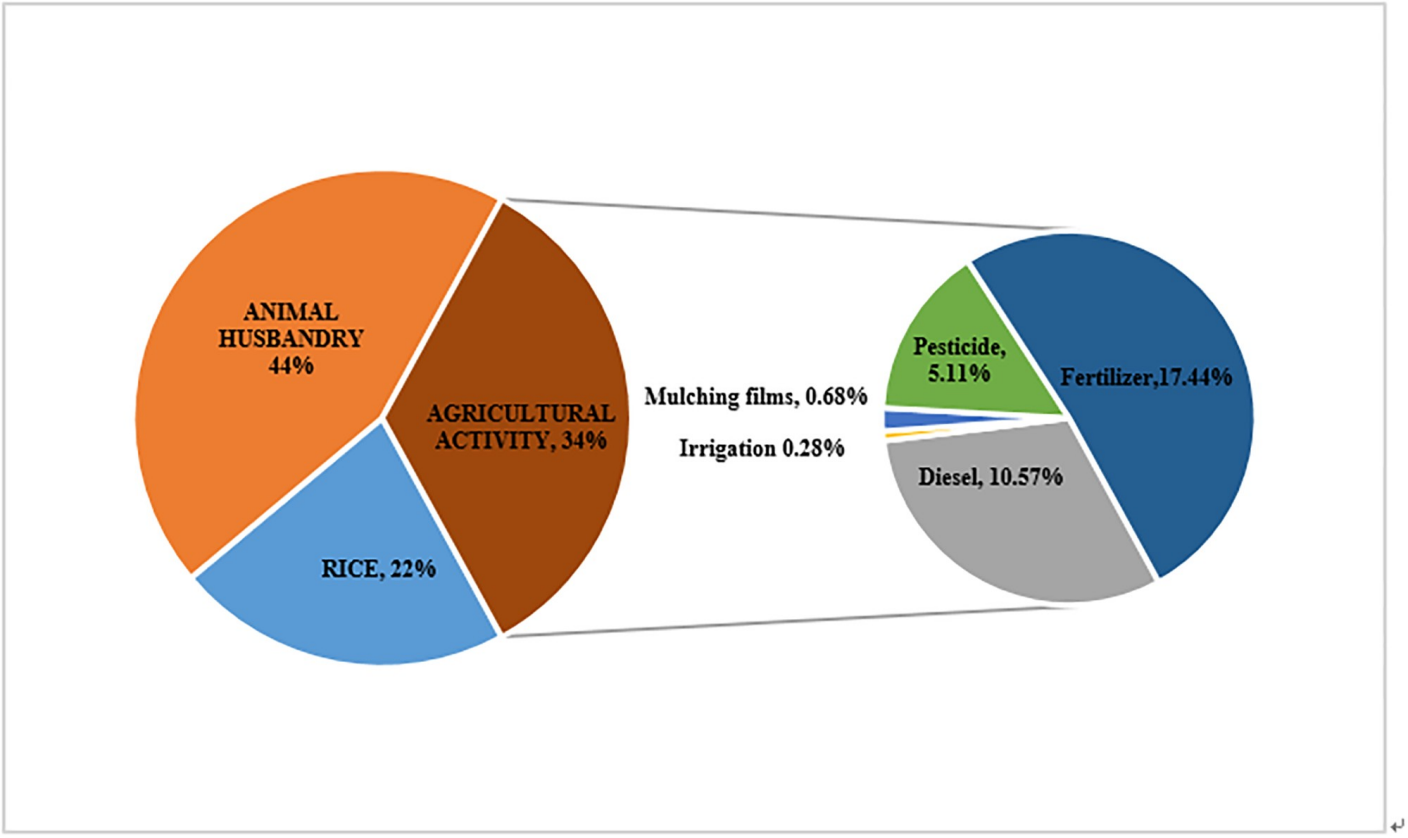


Considering the availability of data and the quality of data, this paper selects the panel data of 31 provinces from 1999 to 2017. The original data are mainly collected from China Statistical Yearbook, China Agricultural Yearbook, China Agricultural data collection and China Rural Yearbook. Through calculation, we can get the carbon emissions generated by agricultural activities, crop production and livestock in 1999–2017. The calculation results are shown in Fig 2. The Fig 3 show average proportion of emissions from each carbon source from 1999 to 2017.

Our results indicate change characteristics of agricultural carbon emissions as show in the Fig 2: the evolution characteristics of China’s carbon emissions show the track of “stable-fluctuation-stable”. After 2008, the carbon emissions have increased steadily. Our results reveal agricultural carbon emissions may have an increasing trend in the future [23].

Based on the above results, we speculate that the following reasons may affect agricultural carbon emissions: First of all, since the subprime crisis in 2008 in the United States, the sharp decline in demand from the United States has led to the bankruptcy of a large number of enterprises in the Pearl River Delta, and a large number of migrant workers in China have lost their jobs and returned home to engage in agricultural production. The population flow leads to the increase of agricultural production activities, which leads to the increase of carbon emissions in 2008. Therefore, the urbanization level is considered as one of the influencing factors of agricultural carbon emissions.

Secondly, it is found that the variation trend of agricultural carbon emissions in China is mainly determined by the carbon emissions from rice growth and livestock activities. There are two inflection points for agricultural carbon emissions in China: The first turning point was in 2003, which was caused by the reduction of rice carbon emissions. In 2003, rice leaf curl disease broke out in China due to abnormal climate, which caused serious losses to rice production in China. This has resulted in a significant reduction in rice production and a sudden change in carbon emissions. Based on this, this paper introduces the degree of natural disasters to measure the change of agricultural carbon emissions. The second turning point was in 2007, the reduction of carbon emissions from animal husbandry led to the reduction of agricultural carbon emissions. Since the end of 2003, the highly pathogenic H5N1 avian influenza has witnessed an unprecedented outbreak, sweeping across Asia and some European countries. In 2005, avian influenza spread widely in China, which led to a significant decline in poultry breeding industry in 2006. Therefore, agricultural carbon emissions in 2006 were greatly reduced. Moreover, our results show that after 2006, the carbon emission of agricultural activities and rice growth in China has increased gradually, but the livestock industry has decreased year by year. The above analysis proves the impact of agricultural structural changes on agricultural carbon emissions.

Through effectively identifying the influencing factors of agricultural emission reduction, we can establish and perfect the rational agricultural emission reduction target system. Based on the analysis of data statistics and literature, this paper selects the influencing factors from agricultural input, social economy and agricultural environment. Then effectively guarantee the realization of the goal of agricultural emission reduction.

## Construction of spatial weight matrix (SWM)

In the paper, we using the simple binary 0–1 space weight matrix as *W*_*ij*_, the space weight model of the research scope is established.

$$W_{ij}=\{\begin{array}{l} 1,\;\text{When}\;\text{regin}\;\text{i}\;\text{is}\;\text{next}\;\text{to}\;\text{region}\;\text{j}\\ 0,\;\text{When}\;\text{regin}\;\text{i}\;\text{is}\;\text{not}\;\text{next}\;\text{to}\;\text{region}\;\text{j} \end{array}$$ (5)

As shown in the formula in (5), the According to the above equation, if the two regions are adjacent, the corresponding weight element value is 1; if the two regions are not adjacent, the corresponding element value is 0. Finally, we normalize the row so that the sum of its elements is 1.

In the above analysis, we calculated the agricultural carbon emissions (CO_2_) of each province, and took this as the dependent variable. Agricultural carbon emissions are affected by many factors, such as economic development, energy consumption structure, industrial structure, urban, rural structure, and technological progress. Referring to the existing research influencing factors, considering the difference between land and population size between each province, we choose the influencing factors from three aspects of agricultural input, social economy and agricultural environment as explanatory variables. Among them, agricultural investment includes rural electricity consumption (E), per capita mechanization level (DL), total amount of fertilizer application (F); social economy involves per capita agricultural gross product (AGDP), proportion of agricultural output value (P), urbanization level (U); finally, we use disaster area (A) to measure agricultural development. Considering the skew distribution, all variables are logarithmically transformed before the input model. Then, the spatial panel data model is used to estimate the factors affecting China’s agricultural carbon emissions and their spatial spillover effects. Establish spatial lag model, such as formula (6). The descriptive statistical analysis of these variables is shown in Table 3.

$$\begin{array}{l} \;\;\text{ln}\;\;CO2_{it}=\alpha +\beta_{0}\sum_{j=1}^{n}W_{ij}\;\text{ln}\;CO2_{it}+\beta_{1}\;\text{ln}\;AGDP_{it}+\beta_{2}DL_{it}+\\ \beta_{3}\;\text{ln}\;F_{it}+\beta_{4}\;\text{ln}\;U_{it}+\beta_{5}\;\text{ln}\;A_{it}+\beta_{6}\;\text{ln}\;P+\beta_{7}\;\text{ln}\;E_{it}+\varepsilon_{it} \end{array}$$ (6)

Where i is the province and t is the time. *W*_*ij*_ is a 0–1 spatial weight matrix and an economic distance weight matrix. *if*_*it*_ refers to individual fixed effect, *tf*_*it*_ represents the time fixed effect.

**Table 3:** Descriptive analysis of variables.

Category	Variables	Unit	Observations	Min	Max	Mean	Standard Deviation
**Dependent variable**	CO_2_	million ton	589	42.1	2238.42	846.86	534.4
**Agricultural input**	DL	Million Kilowatts/per	589	91.5	13353	2507.653	2577
	F	million ton	589	2.5	716	163.78	135.3
	E	TWh	589	0.2	1869.3	175.19	293.47
**Social economy**	AGDP	Ten thousand yuan /person	589	0.037	1	0.246	0.165
	P	%	589	0.0039	0.38	0.13516	0.07
	U	%	589	0.1683	0.9	0.46785	0.16
**Agricultural development**	A	thousand hectares	589	0	7394	1302.926	1127.39

The spatial error models are constructed as in formula (7)

$$\begin{array}{l} \;\text{ln}\;CO2_{it}=\alpha +\rho \sum_{j=1}^{n}W_{ij}CO2_{it}+\varepsilon_{it}\text{+}\beta_{1}\;\text{ln}\;AGDP_{it}+\beta_{2}DL_{it}\\ +\beta_{3}\;\text{ln}\;F_{it}+\beta_{4}\;\text{ln}\;U_{it}+\beta_{5}\;\text{ln}\;A_{it}+\beta_{6}\;\text{ln}\;P+\beta_{7}\;\text{ln}\;E_{it} \end{array}$$ (7)

The spatial Durbin model is constructed as in Eq (8):

$$\begin{array}{l} \;\text{ln}\;CO2_{it}=\alpha +\beta_{0}\sum_{j-1}^{n}W_{ij}\;\text{ln}\;CO2_{it}+\beta_{1}\;\text{ln}\;AGDP_{it}+\beta_{2}\;\text{ln}\;F_{it}+\beta_{3}\;\text{ln}\;P_{it}\\ +\beta_{4}\;\text{ln}\;U_{it}+\beta_{5}\;\text{ln}\;A_{it}+\beta_{6}\;\text{ln}\;DL_{it}+\beta_{7}\;\text{ln}\;E_{it}+\varphi_{it}+\sigma_{1}\sum_{j-1}^{n}W_{ij}\;\text{ln}\;E_{it}\\ \text{+}\sigma_{2}\sum_{j-1}^{n}W_{ij}\;\text{ln}\;AGDP_{it}+\sigma_{3}\sum_{j-1}^{n}W_{ij}\;\text{ln}\;F_{it}+\sigma_{4}\sum_{j-1}^{n}W_{ij}\;\text{ln}\;P_{it}\\ +\sigma_{5}\sum_{j-1}^{n}W_{ij}\;\text{ln}\;U_{it}+\sigma_{6}\sum_{j-1}^{n}W_{ij}\;\text{ln}\;A_{it}+\sigma_{7}\sum_{j-1}^{n}W_{ij}\;\text{ln}\;DL_{it}\\ +if_{it}+tf_{it}+\varepsilon_{it} \end{array}$$ (8)

The spatial Doberman model reflects the impact of local agricultural input, social economy and agricultural development on carbon emission and its spatial spillover effect.

### Spatial auto-correlation test

The first law of geography believes that a certain spatial attribute value on a geographic unit is related to the same spatial attribute value of its adjacent units, the closer the space is, the greater the relevance will be. The attribute value of the geographical space is dependent on the space. The traditional regression model cannot measure the spatial relationship, and the spatial measurement model can well solve the problem of the regression of the spatial dependence of the geography. Taking into account the mobility of pollutant emissions between regions and the diffusion of technological innovations, the influencing factors of agricultural carbon emissions in a region are unavoidably affected by the influencing factors of neighboring regions.

In order to control the spatial auto-correlation effect of dependent variables, spatial panel data model is used to test the influencing factors and spatial spillover effects in this study. Compared with general panel data model, spatial panel data model considers spatial effects including spatial dependence and spatial spillover effect. In contrast to the cross-section model, spatial panel data can capture the individual consistency of spatial units, then effectively avoid the loss of variables and estimation errors.

Before use the spatial analysis, we test the explanatory variables for multiple linearity first, and the results are shown in Table 4: the variance inflation factor (VIF) value of all variables is less than 10, so there is no collinearity problem between variables. In addition, due to the regression of spatial panel data, the problem of multicollinearity can be reduced, which making parameter estimation more effective.

**Table 4:** Collinearity test of variables.

Variables	VIF
**LnDL**	4.36
**LnAGDP**	1.45
**LnF**	4.94
**LnE**	1.45
**LnA**	1.45
**LnP**	2.72
**LnU**	2.79
**The mean values of VIF**	2.73

Before using the spatial model, a series of spatial Lagrangian tests are used to verify the rationality of introducing spatial effects into the general panel data model. This study considers the spatial factors of the 2017 agricultural carbon emission impact model. As shown in Table 5, the test results show that the non-fixed effect model, spatial fixed effect model and time fixed effect model have significant results, reject the original hypothesis, and there is spatial effect in the data sample. The above results show that the spatial panel model is superior to the traditional panel data model without spatial effect. Spatial econometric models should be used to capture the spatial correlation of factors affecting agricultural carbon emissions at the provincial level.

**Table 5:** Estimation results of non-spatial panel model and LM test.

Variables	Mixed estimation model	Individual fixed effect model	Time fixed effect model	Random effect model
**LnAGDP**	0.33(***)	0.2(***)	0.36(***)	0.19(***)
**LnF**	0.27(***)	0.05(***)	0.26(***)	0.077(***)
**LnE**	0.13(***)	-0.06(***)	0.13(***)	-0.02
**LnP**	0.23(***)	0.048(***)	0.18(***)	0.085(***)
**LnU**	-0.93(***)	-0.07(***)	-1.08(***)	-0.09(***)
**LnA**	0.03(***)	0.00	0.03(*)	0.004
**LnDL**	-0.01	0.11(***)	-0.04(*)	0.1(***)
**_cons**	4.75(***)	6.7(***)	4.6(***)	6.5(***)
**R**^**2**^	0.80	0.88	0.92	0.65
**Adj-R**^**2**^	0.80	0.88	0.90	0.62
**LM spatial lag**	40.2(***)	16.8(***)	38.6(***)	0.30
**Robust LM spatial lag**	38.6(***)	0.09	39.65(***)	0.40
**LM spatial error**	3(***)	0.08	39(***)	1.00
**Robust LM spatial error**	50(***)	0.09	42(***)	1.00

**Note**:

***denotes the significance levels of 1%;

**denotes the significance levels of 5%;

*denotes the significance levels of 10%.

### Spatial characteristics of agricultural carbon emissions

Spatial auto-correlation analysis is used to verify whether the selected samples have spatial auto-correlation. This section uses global Moran’s I to test the spatial correlation of agricultural carbon emissions and their influencing factors. *W*_*ij*_ represents a standardized spatial connection matrix. Global Moran’s I calculation formula (9).

$$GlobalMoran\prime sI=\frac{m\sum_{i=1}^{n}\sum_{j=1}^{n}w_{ij}(x_{i}-\bar{x})}{(\sum_{i=1}^{n}\sum_{j=1}^{n}w_{ij})\sum_{i=1}^{n}{\left(x_{i}-\bar{x}\right)}^{2}}$$ (9)

Where m is the number of spaces, *x*_*i*_, *x*_*j*_ represents the space index i, j, respectively. Global Moran’s I ranges from—1 to 1. The closer the calculation result is to 1, the more obvious the spatial clustering area is, the closer it is to—1, indicating that there are more discrete distribution trends in the spatial memory. The value of the Global Moran’s I closer to 1, it shows that the more obvious the spatial clustering area is; The value of the Global Moran’s I closer to -1, it shows that there are more discrete distribution trends in space.

**Fig 4:**
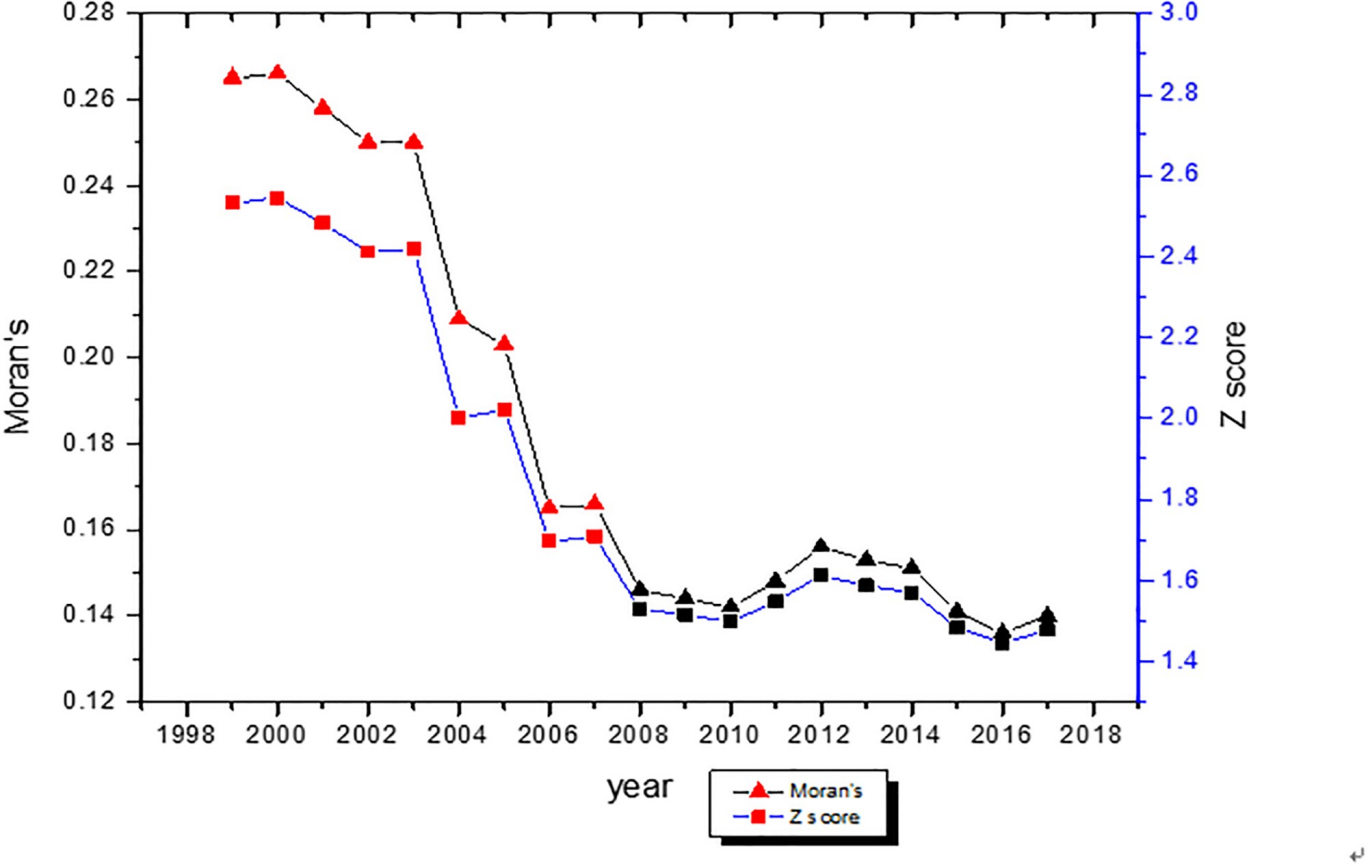


As shown in Fig 4, the Moran’s I value of agricultural carbon emissions is greater than 0. The results show that there is a significant positive auto-correlation relationship between the provincial carbon emissions in China. The value of Moran’s I is positive, means that regions with high agricultural carbon emissions (high group provinces) tend to be distributed together, similar to regions with low emissions (low group provinces). From 1999 to 2017, the global Moran’s I value decreased, indicating that the cohesion trend of China’s agricultural carbon emissions decreased. In general, the spatial auto-correlation test results show that it is necessary to build a spatial panel data model to measure the influencing factors and spatial spillover effects.

**Fig 5:**
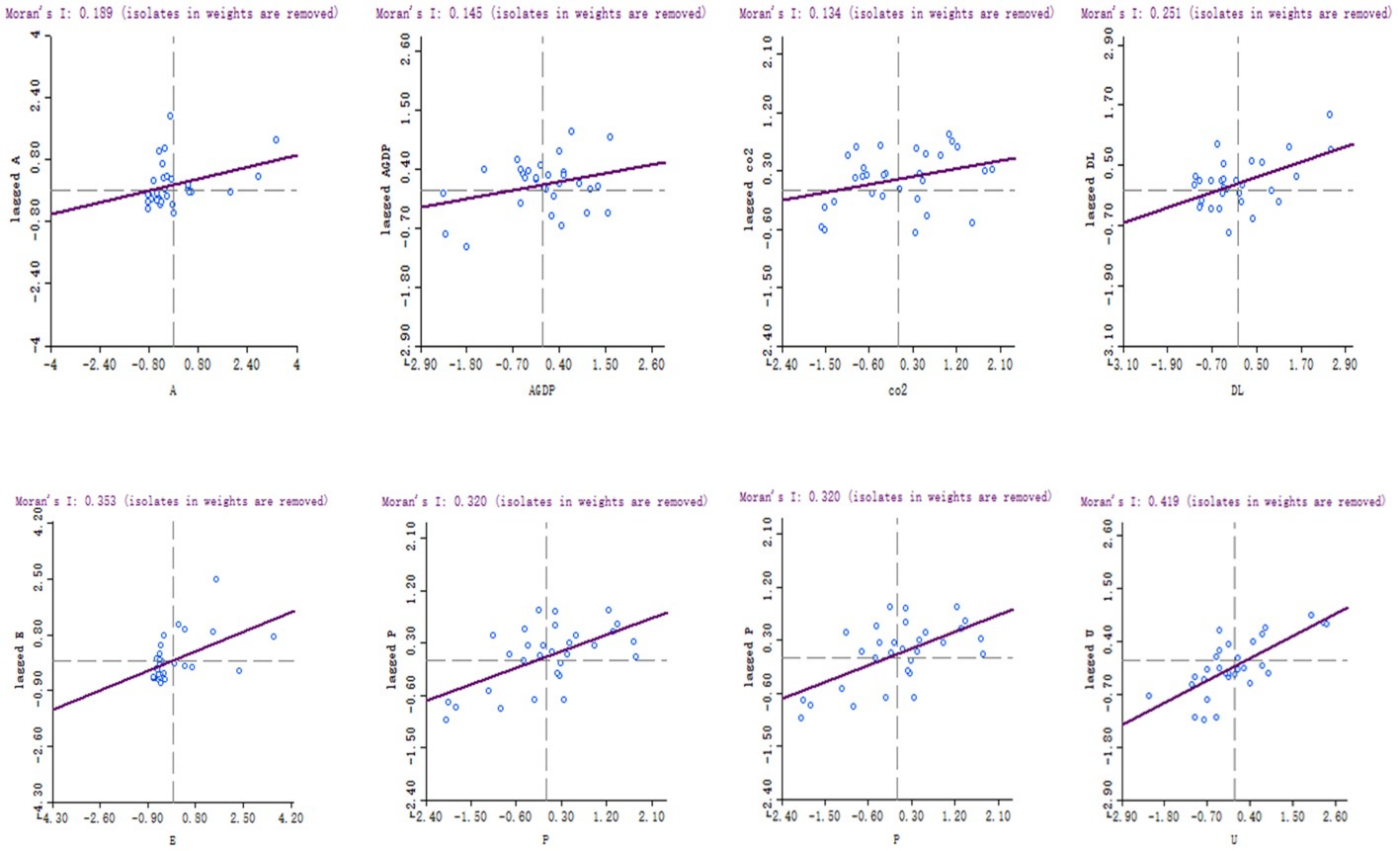


To represent the auto-correlation of different units, Fig 5 shows the Moran’s I scatter distribution of the selected variables in 2017. Each quadrant represents different clustering types: In the first quadrant, HH represents high value points surrounded by similar points; plotted in the second quadrant is LH represents low value points surrounded by high value points; the third quadrant is LL represents low value points surrounded by similar points; in the fourth quadrant, HL represents high value points surrounded by low value points. Moran’s I scatter plot shows that the spatial auto-correlation factors rank U>E>P>DL>F>A>AGDP. Among them, the value of provincial agricultural carbon emission is 0.1304. The statistical results show that most of the points are concentrated in HH and LL clusters. This is another evidence of spatial auto-correlation. It is proved again that the spatial auto-correlation of variables has an important influence on model estimation.

**Fig 6:**
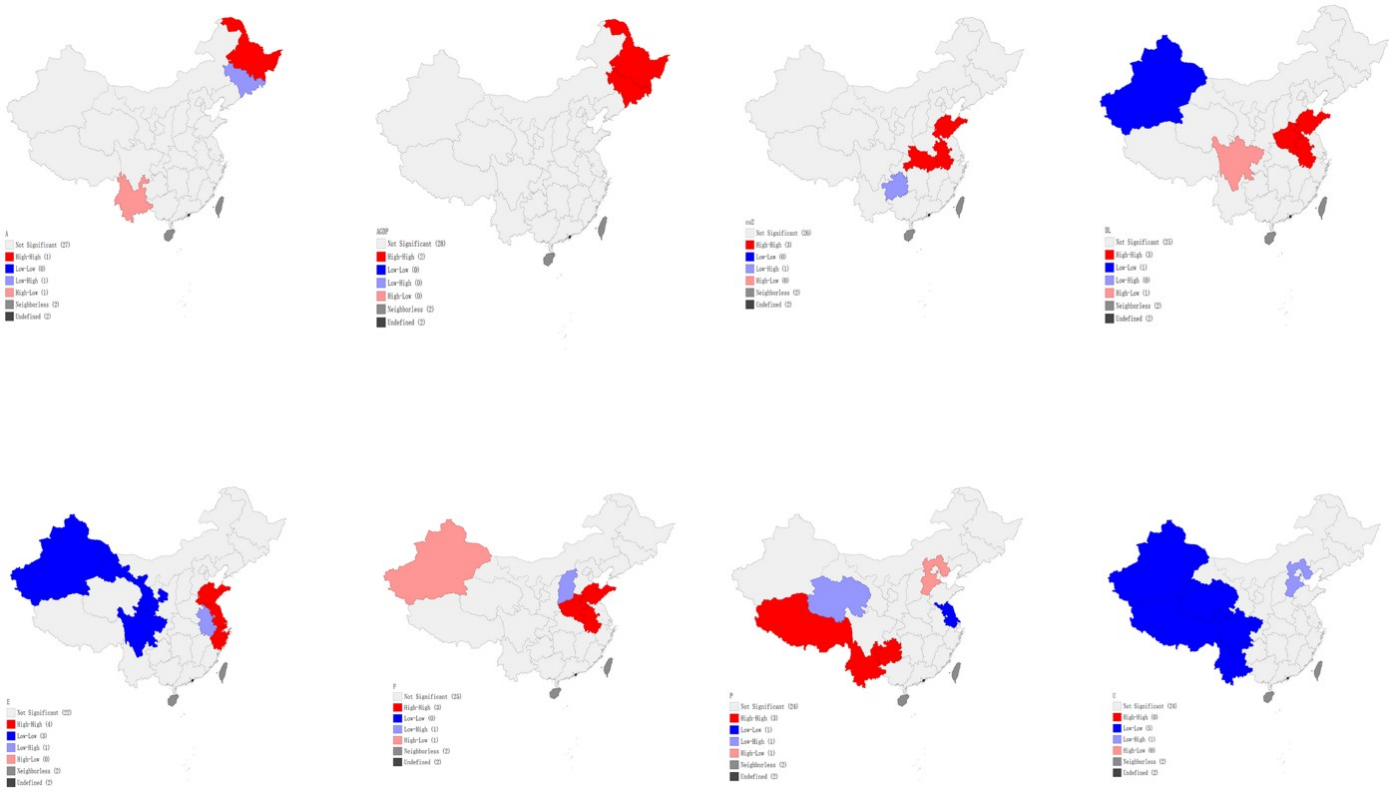


In order to visualize the spatial clustering pattern of each variable more intuitively, Fig 6 shows a specific geographical distribution corresponding to provincial agricultural carbon emissions in 2017. According to Lisa map, 75% of provincials in China’s were in the H-H cluster: Jiangsu, Anhui and Hubei. In these regions, the provincial agricultural carbon emissions were high, and the carbon emissions of adjacent regions were also high. Guizhou Province is in the cluster of L-H. That means although the agricultural carbon emission of Guizhou Province is relatively low, the agricultural carbon emission of its neighboring provinces is relatively high.

According to the cluster diagram of Lisa, independent variables have 39 data points (49% of the total data) in H-H region (these points and the data in the adjacent regions are higher). The data points located in H-H region are mainly concentrated in the agricultural region of north china plain, the middle and lower reaches of the Yangtze river agricultural region, the northeast agricultural region and the southwest agricultural region. These agricultural production areas are mainly plains, which contribute up to 80% of China’s grain production. Secondly, 26% of the areas are in the L-L quadrant, mainly concentrated in Xinjiang and the northwest provinces. The modernization level and economic development of these areas are relatively backward, so the data of these points and adjacent areas are relatively lower. The second quadrant and the fourth quadrant are L-H and H-L respectively. There are 15% of the dependent variables in L-H quadrant, mainly concentrated in Guizhou, Shanxi, Anhui, Jilin, Qinghai and Hebei. The adjacent areas of these regions are mainly coastal provinces and developed cities such as Beijing. Our findings indicate that the level gap between the provinces in L-H region has not been narrowed.

## Results and interpretation

Table 6 shows the estimation results of four spatial panel data models. Random effect model or fixed effect model are selected for each model by Hausman test. If the Hausman statistic is less than 0, it can accept the original hypothesis of random effect (H_0_: Individual effects are independent of regression variables) [23]. In order to further verify the results of model selection, GPM, SAR, SEM, SAC and SDM models are estimated and compared. The experimental results show that for GPM model, SAR model and SAC model, the value of the Hausman is more than 0, The results show fixed effect model is more suitable than the random effect model, while for SEM and SDM the random effect model is more effective than the fixed effect model. Because the overall R^2^ within estimation of SDM model is higher than other models, it can be considered as a relatively good regression model. Therefore, in the following analysis, we mainly explain the influencing factors according to the estimation results of SDM random effect model.

**Table 6:** Spatial panel data model estimation results.

Variable	GPM(FE)	SAR (FE)	SEM (RE)	SAC (FE)	SDM (RE)
**lnAGDP**	0.15 (***)	0.118(***)	0.16(***)	0.16(***)	0.15 (***)
**lnF**	0.05 (***)	0.045(***)	0.05(***)	0.038 (***)	0.041(***)
**lnE**	-0.011	-0.06	-0.0164	-0.017(*)	-0.012
**lnDL**	0.1(***)	0.08 (***)	0.1(***)	0.1 (***)	0.086(***)
**lnA**	-0.005	-0.0041	-0.005	-0.0046(*)	-0.004
**lnP**	0.08(***)	0.058 (***)	0.076 (***)	0.06 (***)	0.03 (*)
**lnU**	-0.024	-0.02	-0.024	-0.008	-0.04 (*)
**constant**	6.8 (***)		6.63(***)		3.6 (***)
**lag lnA**					0.011
**lag lnP**					-0.18 (***)
**lag lnE**					-0.18 (***)
**Statistics**					
**R**^**2**^ **between**	0.6	0.32	0.6	0.52	0.13
**R**^**2**^ **overall**	0.6	0.3	0.52	0.5	0.13
**R**^**2**^ **within**	0.31	0.3	0.3	0.3	0.4

**Note**:

***denotes the significance levels of 1%;

**denotes the significance levels of 5%;

*denotes the significance levels of 10%.

### Analysis of direct effects

Table 7 calculates the influencing factors of agricultural carbon emissions at provincial level in China. The results show that the direct average effect coefficients of AGDP, F, E, U and DL are 0.16, 0.043,—0.04,—0.041 and 0.1, respectively. In other word, when AGDP, F and DL increase by 1%, agricultural carbon emissions will increase by 0.16%, 0.044% and 0.1%, respectively. The above data indicate that the AGDP is the direct driving factor of agricultural carbon emissions, followed by DL. Although the use of agricultural fertilizer has a certain effect in promoting agricultural carbon emissions, it has a smaller effect compared with the former two.

**Table 7:** The direct, indirect, and total effects of explanatory variables.

Variable	Effects	SDM (RE)	SAC (FE)	SAR (FE)
**lnAGDP**	Average direct effect	0.16(***)	0.16(***)	0.13(***)
	Average indirect effect	0.14(***)	-0.05(***)	0.114(***)
	Average total effect	0.32(***)	0.12(***)	0.24(***)
**lnF**	Average direct effect	0.043(***)	0.04(***)	0.047(***)
	Average indirect effect	0.04(***)	-0.01 (***)	0.042(***)
	Average total effect	0.083(***)	0.03(***)	0.09(***)
**lnE**	Average direct effect	-0.04(***)	-0.016	-0.033(***)
	Average indirect effect	-0.33(***)	0.0046	-0.03(***)
	Average total effect	-0.37(***)	-0.012	-0.066(***)
**lnDL**	Average direct effect	0.1(***)	0.1(***)	0.088(***)
	Average indirect effect	0.08(***)	-0.03(***)	0.078(***)
	Average total effect	0.18(***)	0.07(***)	0.166(***)
**lnA**	Average direct effect	-0.003	-0.0046	-0.0044
	Average indirect effect	0.017	0.0013	-0.0041
	Average total effect	0.014	-0.0033	-0.0085
**lnP**	Average direct effect	0.008	0.063(***)	0.065(***)
	Average indirect effect	-0.3(***)	-0.02(***)	0.06(***)
	Average total effect	-0.29(***)	0.043(***)	0.125(***)
**lnU**	Average direct effect	-0.041(*)	-0.01	-0.0225
	Average indirect effect	-0.34	0.0029(***)	-0.02
	Average total effect	-0.075(*)	-0.0071	-0.04

**Note**:

***denotes the significance levels of 1%;

**denotes the significance levels of 5%;

*denotes the significance levels of 10%.

Contrary to the above variables, the direct effect of rural electricity consumption and urbanization on agricultural carbon emissions is—0.04%, at a significant level of 1%. The data show that when the power level and urbanization level increase by 1%, the agricultural carbon emissions will be reduced by 0.04%. The increase of agricultural electric power use and population aggregation to cities may directly inhibit agricultural carbon emissions in China. The model shows that increasing power supply and accelerating urbanization are effective ways to reduce agricultural carbon emissions. Feedback effect refers to that the explanatory variables of a certain region will affect the explanatory variables of “neighboring” areas, which in turn will affect the explained variables of the region. The feedback effect of E is -0.14, which means that affected by neighboring provinces, the feedback effect can reduce the agricultural carbon emissions of the original provinces. The feedback effect of U is -0.001, which means that affected by neighboring provinces, the feedback effect can reduce the agricultural carbon emissions of the original provinces.

### Space spillover effect

Compared with the average direct effect and the estimated coefficient, the average total effect can reflect the actual effect of the influencing factors more comprehensively. The total positive effects of AGDP, F and DL on agricultural carbon emissions are 0.32, 0.0083 and 0.18 respectively. On the contrary, P, E and U have inhibitory effects on agricultural carbon emissions, with coefficients of -0.29, -0.37 and -0.075, respectively. Among them, the average total effect of P mainly comes from the spatial spillover effect. Due to the influence of spatial spillover effect in the results, it is likely to distort the actual effect of the influencing factors by using the estimated coefficients in Table 4 for analysis, directly. So the total, direct and indirect effects among variables should be further calculated. Among the influencing factors we explored, per capita agricultural GDP can be considered as the main driving factor of agriculture. Considering the population factor, with the continuous growth of social economy and the increase of per capita agricultural GDP, China’s agricultural carbon emissions will inevitably continue to grow. The spatial spillover effect of AGDP is 0.14% (at a significant level of 1%), which indicates that the increase of AGDP in the province will not only promote the growth of agricultural carbon emissions in the original province, but also promote the increase of agricultural carbon emissions in neighboring provinces. In addition, the use of agricultural fertilizer and the degree of agricultural machinery will also increase agricultural carbon emissions. Machinery substitutes for labor, which increases the consumption of diesel, electricity and other energy sources, leading to the increase of agricultural carbon emissions. At the same time, the research results show that the increase of per capita agricultural GDP and rural electricity consumption can not only increase the carbon emissions of the province, but also affect the carbon emissions of neighboring provinces.

The results show that the increase of rural electricity consumption, urbanization level and the proportion of agricultural output value will reduce agricultural carbon emissions. Among them, rural electricity consumption has the maximum average elasticity of total effect, and its total effect is less than 0. It can be considering that increasing rural electricity consumption is the main way to reduce agricultural carbon emissions. According to the data survey, the proportion of new energy power generation in China has increased from 17.8% in 2000 to about 30% in 2017. China’s agricultural activities are mainly based on electricity and energy. Therefore, the increase of renewable energy power generation can directly reduce agricultural carbon emissions. At the same time, the research shows that the total effect of rural electricity consumption mainly comes from the spatial spillover effect, it shows that compared with the original provinces, the increase of rural power consumption has more obvious impact on neighboring provinces.

The increase of urbanization level can reduce agricultural carbon emissions, mainly through direct effects on agricultural carbon emissions, compared with the effect of the original provinces, the increase of rural power consumption has more obvious effect on the emission reduction of neighboring provinces. The increase of urbanization level can reduce agricultural carbon emissions, mainly through the direct effect on agricultural carbon emissions. As the flow of rural population has led to the decrease of agricultural workers, moreover, the growth of agricultural production materials such as chemical fertilizer and pesticide has slowed down or negative growth, which has led to the reduction of agricultural carbon emissions. The proportion of agricultural output value in the primary industry mainly affects the agricultural carbon emissions through spillover effect. The proportion of agricultural output value in the primary industry mainly affects the agricultural carbon emissions through spillover effect. This study once again confirmed the emission reduction effect of agricultural economic structure, so we should further play its role in regional emission reduction. The proportion of traditional agriculture should be further reduced in the eastern developed areas where the proportion of agriculture is relatively low, give more attention to central and western regions with more comparative advantages. Meanwhile, strengthen the division and cooperation of agricultural development, and supporting facilities for agricultural industry in different provinces. Develop advantageous and characteristic agriculture according to local conditions.

## Conclusions and policy implications

As one of the largest carbon dioxide emission sectors, the agriculture sector has the responsibility to find ways to reduce carbon emissions and achieve sustainable development. The identification of the impact factors on CO_2_ emissions is critical for reducing CO_2_ emissions in agriculture sector. Several studies have demonstrated the impact of economic factors on agricultural emissions, particularly in developing countries. However, this study did not fully consider the spatial and temporal effects of different factors.

The identification of agricultural input, economic development and agricultural environmental factors is the first step to reduce carbon dioxide emissions and achieve sustainable development goals. In order to accurately estimate agricultural input, economic development and agricultural environment, the significance of the impact factors on agricultural carbon emissions. In our research, strictly determine the model specification, based on the five models of GPM, SAR, SEM, SAC and SDM, we chose the spatial panel data of 31 provinces in China from 1999 to 2017. The paper calculates the spatial factors that affect China’s agricultural carbon emissions, and proves the direct utility and spatial spillover factors of agricultural development factors on agricultural carbon emissions. Through the comparative analysis of the models, it is proved that economic factors and agricultural input factors have direct effects and spatial spillover effects on agricultural carbon emissions except for agricultural environmental factors. The results show that: the indirect impact is mainly manifested in per capita agricultural output value, per capita total mechanical power, rural electricity consumption and agricultural structure; the direct impact is per capita agricultural output value, total mechanical power, rural power consumption and urbanization level. It is worth noting that agricultural input factors play an increasingly important role in reducing CO_2_ emissions. Economic development is still considered to be one of the drivers of environmental pressure. In the short term, strengthening environmental protection may bring some pressure to the economic development of some places, but to achieve high-quality development, we must fundamentally solve the problem of environmental pollution. The development of agriculture should combine economy with environment.

Following the above conclusions, some policy implications may emerge automatically. The significance of spatial auto-correlation of agricultural carbon emissions means that the emission reduction decision-making unit should be expanded from within the region to the inter region. From the theoretical point of view, the cross regional impact cannot be ignored in the evaluation of agricultural CO_2_ emission level. In addition, the existence of radiation spatial auto-correlation indicates that the influence of sequence lag should also be considered in the evaluation of regional or adjacent regional level. Joint decision-making among regions is an effective way to achieve the goal of regional overall emission reduction.

According to the results of all the models, except economic factors, agricultural input has significant positive spillover effect on CO_2_ emissions. Among them, the total power of agricultural machinery per capita has more obvious effect on CO_2_ emission than the use of agricultural chemical fertilizer. Under the advocacy policy of low-carbon agriculture in China, the effect of agricultural fertilizer on CO_2_ emission has achieved certain effect. But the total power of agricultural machinery is the comprehensive influence of other factors, such as population, economy and technology. It is not only an important embodiment of agricultural comprehensive production capacity, but also an important symbol of agricultural modernization. “The guiding opinions on accelerating the transformation and upgrading of agricultural mechanization and agricultural machinery equipment industry” issued by the State Council in 2018, clearly states that the total power of agricultural machinery in China will exceed 1 billion kilowatts, and the comprehensive mechanization rate of crop cultivation and income in China will reach 70%, by 2020. The level of Agricultural Mechanization in China has been improved unprecedentedly. However, while vigorously developing agricultural mechanization, we should also guard against the negative impact of agricultural mechanization and the increase of agricultural carbon emissions caused by agricultural mechanization. Based on this, China’s agricultural mechanization should speed up the development in the direction of large-scale compound, energy-saving and efficient, intelligent and accurate, so as to improve the scale of agricultural production in China.

The increase of rural electricity consumption, urbanization level and agricultural structure will reduce CO_2_ emissions. The level of urbanization mainly affects carbon emissions through direct effects, while the agricultural structure mainly affects agricultural carbon emissions through spatial spillover effects. It must be noted that urbanization directly affects carbon emissions through population migration, which directly leads to the reduction of Chinese farmers and will cause hidden dangers to China’s agricultural development. The role of agricultural power consumption and agricultural structure in emission reduction is based on the development of new energy and technology. Therefore, the state can promote energy-saving technology and new energy power generation technology, develop energy-saving and emission reduction technologies for grain production, and strengthen the deep integration of agricultural machinery and agronomy, so as to strengthen the inhibitory effect of Technological Development on agricultural carbon emissions. Actively expand the implementation scope of “climate smart agriculture project”, so as to realize the increase of grain production and emission reduction in China.

## Supporting information

S1 Data (XLSX)
